# A continuum of languishing to flourishing: exploring experiences of psychological resilience in multiple sclerosis family caregivers

**DOI:** 10.1080/17482631.2022.2135480

**Published:** 2022-11-04

**Authors:** Odessa McKenna, Afolasade Fakolade, Katherine Cardwell, Lara A. Pilutti

**Affiliations:** a Faculty of Medicine, University of Ottawa, Ottawa, ON, Canada; b School of Rehabilitation Therapy, Queen’s University, Kingston, ON, Canada; c Interdisciplinary School of Health Sciences, University of Ottawa, Ottawa, ON, Canada; d Brain and Mind Research Institute, University of Ottawa, Ottawa, ON, Canada

**Keywords:** Resilience, family caregivers, multiple sclerosis, interviews, thematic analysis

## Abstract

**Purpose:**

Resilience research in family caregiving in chronic neurological conditions is growing, but multiple sclerosis (MS) caregivers are noticeably absent from this body of work. MS caregivers represent a unique population due to the disease’s early onset, prolonged life expectancy, and heterogeneity. As such, this study aimed to explore MS caregivers’ conceptualizations of resilience, examine MS caregivers’ experiences of resilience development, and determine which assets and resources influence resilience in this role.

**Methods:**

Twenty-four Canadian MS caregivers were recruited. Semi-structured in-depth interviews were conducted with questions derived from an ecological resilience framework. Data were analysed using reflexive thematic analysis.

**Results:**

Themes constructed a cyclical resilience model, beginning with encounters with hardship and extending to thriving adjustment. Subthemes included reports of additive challenges, impactful individual and community resources, and multi-level adaptive pathways. Within this cycle, the achievement of healthy adjustment exerted a positive feedback function and informed future responses to lifelong challenges.

**Conclusions:**

Despite the salience of resilience processes within caregiver testimonies, inadequate resources at societal levels were evident. These findings afford researchers and decision-makers relevant information for designing and implementing resilience-building interventions for MS caregivers that attend to contextual factors and current systemic support deficiencies.

## Introduction

Multiple sclerosis (MS) is a complex chronic disease estimated to affect over 2.8 million individuals worldwide (Walton et al., 2020). MS is an immune-mediated, neurodegenerative disease involving demyelination of the central nervous system. In most persons affected by MS, the initial clinical course (relapsing-remitting MS) is characterized by defined clinical episodes of neurological symptom exacerbation, followed by residual deficits or recovery, and stable neurological disability between episodes (Lublin et al., 2014; Thompson et al., 2018). A minority (approximately 15%) of individuals present with a gradual accumulation of neurological disability from the onset (primary progressive MS), typically independent of relapses (Lublin et al., 2014). It is estimated that over half (up to 70%) of people with an initial relapsing MS course will transition to a course of gradual worsening without defined relapses (secondary progressive MS) within roughly 10–20 years of disease onset (Scalfari et al., 2014; Thompson et al., 2018).

Across all disease subtypes, MS is characterized by increasing impairment over time. Despite disease severity, the life expectancy of persons with MS has increased significantly over the past two decades (Sanai et al., 2016), likely attributable to improvements in treatments, understanding of comorbidities, and multidisciplinary integrated medical care models (Marrie, Donkers et al., 2022). MS is now considered a life-long disease, subject to the co-occurrence of natural ageing processes (Awad & Sütve, 2010; Motl et al., 2021). Due to its early onset in young-adult life, increasing life expectancy, and unpredictable disease course, MS carries with it great physical, psychological, and social costs for the person with the disease, as well as their proximate friends and families who must also navigate its ramifications over the extended life course (Feigin et al., 2019).

### Family caregiving in multiple sclerosis

As the disease progresses, the majority of persons living with MS experience the accumulation of permanent long-term disability (Katrych et al., 2012), which results in the loss of functional independence and the increased need for personal assistance (Hlabangana & Hearn, 2020; McKenzie et al., 2015). The support needed by persons with MS is largely provided by unpaid community-dwelling caregivers, usually friends or family members (Rajachandrakumar & Finlayson, 2022). These family caregivers undertake a variety of services which offer essential forms of support (e.g., physical, emotional, informational) to enable persons with MS to participate in daily life activities and remain functioning within their home amidst gradual disease progression (Hughes et al., 2013). As a result, family caregivers may experience role overload, physical taxation, and financial strain due to lost income (Hlabangana & Hearn, 2020). Also, family caregivers may lack knowledge regarding the complex needs of the care-recipient (Capistrant, 2016), posing additional challenges. Consequently, the magnitude and extent of caregiving duties may result in poor mental, emotional, and physical health outcomes (Legg et al., 2013). In fact, MS caregivers report increased levels of stress, depression, anxiety, social isolation, and poorer quality of life in comparison to non-caregiver age-matched controls (Topcu et al., 2016).

Nevertheless, the experience of caregiving for a loved one with MS is seldom uniform (Bekhet & Avery, 2018). Evidence suggests that MS caregivers report a wide range of benefits associated with their role, such as personal growth, strengthened relationships, deepened empathy, and an increased sense of appreciation for life (Fave et al., 2017; Shim et al., 2012). The coexistence of both benefits and hardships within the MS caregiving role implies that, in many instances, individuals have the means to adapt to the demands of caregiving despite potential for significant stressors. This evidence highlights the need for strategies to reduce burden and leverage positive adaptations in MS caregivers (Buchanan et al., 2011). However, before effective strategies can be developed, we must understand precisely which factors account for differences in experiences of adaptation to the caregiving role. One factor that may be responsible for variability in the caregiver experience is psychological resilience (Bekhet & Avery, 2018).

### Psychological resilience

The concept of resilience is lacking a unified theoretical foundation (McKenna et al., 2022), with two prominent conflicting schools of thought: some perceive resilience as a fixed individual trait, while others define resilience as a dynamic process (Windle & Bennett, 2011). Still, most scholars agree on three core components of resilience: 1) an encounter with adversity; 2) adaptation; and 3) the achievement of healthy functioning (Donnellan et al., 2015). Specifically, within the context of caregiving, resilience is conceptualized as the maintenance of well-being despite experiencing high care demands (Joling et al., 2016).

Moreover, the process of resilience is believed to depend on diverse resources from multiple levels, which interact with one another (Windle & Bennett, 2011). Informed by ecological theory, Windle and Bennett (2011) developed a theoretical resilience framework for family caregivers to better delineate the mechanisms by which resilience operates across inter-related ecological levels. Multiple studies (Bennett et al., 2016; Donnellan et al., 2015; Donnellan et al., 2021; Han et al., 2019) have utilized this framework to comprehend caregiver resilience as influenced by interplaying physical, social, and environmental contexts. This framework recognizes that caregivers draw on individual (i.e., psychological, biological, health behaviours, monetary materials), community (i.e., social support, social cohesion, participation, neighbourhoods, housing), and societal (i.e., policies, government agencies, health systems, social services) resources, which may enhance risk for caregiver burden, or alternatively, foster resilience (Han et al., 2019; Windle & Bennett, 2011). This framework emphasizes the multidimensional nature of resilience as it may be cultivated within the individual and the broader social context (Donnellan et al., 2015; Windle & Bennett, 2011).

### The current study

In MS, the early age of symptom onset (20–40 years; Walton et al., 2020), and comparable life expectancy to someone without the disease (Lunde et al., 2017), means that the MS caregiver role can encompass a lifetime, spanning several key life stages, such as parenthood and career-building (Topcu et al., 2016). Recent grounded theory work has sought to remodel life course perspectives in persons living with MS (Satinovic, 2017), drawing from studies of healthy ageing in this population (Ploughman et al., 2012). Findings from this qualitative work (Satinovic, 2017) depicted the MS life course as a complex and staged process of transformation with the primary objective of living a life with MS as well as possible. To echo this perspective among family caregivers coexisting alongside persons with MS over the lifespan, the role of MS caregiving may be inclusive of numerous life periods and may be compounded by additional important life roles (e.g., as a parent, professional, family patriarch, community member). Thus, the MS caregiving experience corresponds with expansive life challenges and stressors and is, in turn, very different from the more commonly studied caregiving populations of older adults and care-recipients with other chronic neurological conditions (CNCs) which typically emerge later in life (e.g., dementia). Understanding the unique experiences of MS caregivers is an imperative step towards the development of tailored strategies, programs, and interventions that may begin to address the unique support needs of this population. Specifically, via the application of the ecological resilience framework for caregivers (Windle & Bennett, 2011), the aims of this study were to: a) explore MS caregivers’ conceptualizations of resilience; b) examine how MS caregivers develop and experience resilience; and c) determine which assets and resources influence resilience in MS caregivers.

## Method

### Study design

The present study used a qualitative design consisting of semi-structured interviews. This design was selected because such interviews are conducive to eliciting rich narratives of caregivers’ own unique experiences, enabling exploration of perspectives, beliefs, and the role of relationships within the context of resilience cultivation (Kitter & Sharman, 2015). This study was framed by ontological relativism and epistemological constructivism. We acknowledge that multiple context-dependent realities exist, and that knowledge is socially constructed based upon unique personal experiences and values (Levers, 2013). Guided by these philosophical assumptions, we elected to focus on individuals’ perceptions through semi-structured interviews exploring subjective experiences of resilience. We sought rich depictions of each participant’s experience and worked to generate an understanding of resilience that also provided room for variations.

### Population and measures

Participants were recruited from a longitudinal online survey study of resilience in MS family caregivers conducted by our research team. The inclusion criteria for the current study were: (i) 18 years or older; (ii) currently providing physical, emotional, or informational assistance for a family member or close friend with MS; (iii) a resident of Canada; (iv) and fluent in English. Individuals who reported difficulty with memory, calculation, or reasoning that significantly interfered with their daily functioning were excluded. Baseline survey participants who expressed interest in the interview component (*n* = 441) were contacted via email. Out of those contacted, most (*n* = 417) did not respond further. A total sample of 24 participants was obtained.

Demographics, caregiving characteristics, and resilience levels were captured quantitatively in the initial online survey. Demographic information included age, biological sex, race, relationship to care-recipient, marital status, education, employment status, and annual household income. We asked caregiver participants to report the level of MS-related disability of their care-recipient using the Patient-Determined Disease Steps (PDDS) scale (Hohol et al., 1995), which has demonstrated good psychometric properties (Marrie, McFadyen et al., 2022). The PDDS uses a nine-level ordinal scale (0 = *normal* to 8 = *bedridden*) to report neurological disability level. Additionally, years of caregiving, and the average minutes per day spent in caregiving activities were collected. Resilience was measured using the 25-item Connor-Davidson Resilience scale (CD-RISC; Connor & Davidson, 2003). This instrument is based on a five-point scale (0 = *not true at all* to 4 = *true nearly all the time*) over the span of the previous month. Total scores range between 0 and 100, with higher scores reflecting greater resilience levels. This scale has demonstrated good consistency, reliability, and validity (Connor & Davidson, 2003; Windle et al., 2011).

Table I provides a summary of caregiver characteristics and mean resilience scores. Participants (*n* = 24) were mostly middle aged, with just over half reporting as female. Most caregivers in the sample were spouses. With respect to the disability level of the care-recipient with MS, participants reported a median PDDS score of 5 (i.e., unilateral support required to walk 25 feet). The duration of caregiving ranged from one to 30 years. The amount of time per day dedicated to providing assistance ranged from 10 minutes to 10 hours. The mean CD-RISC score was 67.0.

**Table I.** Demographic information and resilience levels of participants.

n = 24	Mean	Min	Max
Age	56.9	31	80
Duration of Assistance (in years)	13.7	1	30
Minutes of Assistance Per Day	183.8	10	600
CD-RISC^a^	67.0	52	94
	**Median**	**Min**	**Max**
PDDS^b^	5	0	7
	**n**		
Sex			
Male	11		
Female	13		
Race			
European descent	11		
North American descent	11		
Other Asian descent	1		
Unknown	1		
Relationship to Person with MS			
Spouse/Common Law partner	20		
Parent	2		
Adult child	1		
Sibling	1		
Marital Status			
Married/Common Law	21		
Separated/Divorced	2		
Widowed	1		
Highest Education Level			
High school or equivalent	3		
Technical or trade school	2		
College	1		
Bachelor’s degree	8		
Master’s degree	7		
Doctoral degree	2		
Unknown	1		
Employment Status			
Employed full time	8		
Unemployed	4		
Retired due to age or life course decision	11		
Retired due to medical reasons	1		

^a^Connor-Davidson Resilience (CD-RISC) scale total score.

^b^Patient Determined Disease Steps (PDDS) score of 6 is a threshold for walking 25 feet with bilateral support.

### The interview process

Following a synthesis of current caregiver and resilience literature referenced below, a semi-structured interview guide was developed (see Appendix 1). The guide was based on questions utilized in a qualitative focus group study designed to examine resilience and related factors in MS caregivers and other MS stakeholders (Silverman et al., 2017), in addition to recent qualitative applications (Donnellan et al., 2017, 2019; Han et al., 2019) of the ecological resilience framework for caregivers (Windle & Bennett, 2011). Further, the interview guide was formulated to acknowledge the global COVID-19 pandemic context during which the interviews took place. However, in alignment with the aims of the present study, this manuscript reports solely on general resilience-related experiences pertaining to pre-pandemic or general circumstances.

Interviews were conducted between July and October of 2020. Interviews were conducted primarily by the first author, with collaborative support from the second author, who both identify as women. The first author possesses experience interacting with persons with MS and their families in a health research setting and received further training for this role under doctorate-level researcher supervision. The second author is highly skilled in qualitative methods and has considerable research experience working with persons with neurological disabilities and their family caregivers. The authors had previous contact with some participants in research or community settings; there were no established relationships with participants in healthcare professional roles.

Interviews took place primarily by videoconference (Zoom Video Communications Inc, San Jose, California; *n* = 22) or telephone (*n* = 2), according to participant preference. While face-to-face in-person interviews are commonly preferred for building rapport and attending to nonverbal cues (Carr & Worth, 2001), this was not possible given that this research was conducted during the COVID-19 pandemic when physical distancing and other restrictions largely prevented in-person interactions. Fortunately, research comparing the use of remote interview methods with face-to-face interviews has demonstrated no differences in the resulting data (Archibald et al., 2019; Sturges & Hanrahan, 2004; Trier-Bieniek, 2012). Indeed, remote communication can have added benefits, such as increased participant comfort and anonymity, decreased social pressures, and increased access to hard-to-reach groups facing accessibility limitations (Sturges & Hanrahan, 2004). Furthermore, the interviewer remained attentive to non-verbal cues as participant faces are visible on videoconference, and cues such as pauses and changes in intonation are present when speaking on the phone. Via these two interview platforms, the interviewer was able to provide an encouraging, affirming, and non-judgemental space, which promoted detailed and rich recollections of past experiences (Jones et al., 2019), despite the sensitivity of content. Interviews were audio‐recorded, transcribed, and anonymized. Interviews lasted between 45 and 90 minutes.

### Data analysis

Interview transcripts were entered into NVivo 12 (QSR International Pty Ltd, Melbourne, Australia) for coding by the first author. A six-step reflexive thematic analysis was conducted (Braun & Clarke, 2006; Braun et al., 2016). In accordance with recent developments in qualitative health research (Braun & Clarke, 2021), data saturation was not determined in advance of analysis because this goal is not consistent with the values of reflexive thematic analysis. Themes were situated within the context of caring relationships and MS as a socially constructed illness, informed by how individuals come to live and comprehend illness and how illnesses are formulated by sociocultural factors (Conrad & Barker, 2010; Davies et al., 2015). Additionally, our approach used the ecological resilience framework as a tool to contextualize and deepen the interpretation of themes. In acknowledgement of the global COVID-19 context during which data were collected, themes specific to the COVID-19 pandemic were not included in this analysis and this manuscript reports on general resilience experiences pertaining to pre-pandemic circumstances.

The first step of analysis consisted of data familiarization by the first author and was accomplished by actively reading and re-reading the material. In the second phase of analysis, the first author generated an initial codebook consisting of both deductive and inductive patterns of meaning organized across the three ecological levels. The existing ecological resilience framework was used to code themes in relation to caregivers’ encounters with adversity, individual, community, and societal resilience resources, and adjustment consequences. This form of organization enabled the identification of patterns among resilience processes within the dataset with respect to their ecological location, constituent similarities, and manner of interaction with other emergent resilience processes which permeated across ecological levels.

As new codes emerged more inductively, the codebook was expanded and refined. The second and last authors acted as “critical friends” to challenge initial codes, contribute to the development of new codes, and stimulate reflection. A robust set of codes representing relevant elements of resilience was finalized during the second round of coding. Once coding was completed, the first author entered the third phase of analysis and began identifying cardinal themes by examining high-level patterns within and beyond the ecological framework. In the fourth step, candidate themes were reviewed to ensure that there was clarity in scope and boundary of each theme. A thematic map was generated to demonstrate a relationship between themes and subthemes. The fifth phase involved final discussions between all authors, where overarching themes were inferred, and all themes were explicitly defined via text. In the final phase, the entire dataset was reviewed to ensure that the candidate themes were representative, and that themes demonstrated an overall fit with the research objectives and supporting literature.

### Study quality

Aligning with our relativist approach, we did not view quality criteria for this research as universal or concrete because values for quality are ever-changing and highly context dependent (Sparkes & Smith, 2014; Tracy & Hinrichs, 2017). Rather, we drew from a dynamic list of relevant traits from different scholars which have markedly contributed to the qualitative methodological landscape (Braun & Clarke, 2006; B. Smith, 2018; Tracy & Hinrichs, 2017). Ultimately, we chose the following criteria: the worthiness of the topic (e.g., public health significance of caregiving across disciplines); resonance (e.g., thick descriptions and rich interpretations of the data that could be transferable to different situations); plausibility (e.g., situating our findings in relation to existing frameworks of resilience); meaningful coherence (e.g., compatibility between the study purpose, methods, results, and interpretation); and reflexivity (e.g., multiple critical friend discussions to stimulate reflection upon, and exploration of alternative explanations and interpretations of the data).

### Ethical considerations

Both written and verbal information about voluntary participation, confidentiality protocols, study aims, and data collection processes were provided to participants prior to each interview. Due to the remote nature of this research, participants provided verbal informed consent to the interviewer, which was documented via audio recording. To assure participants’ confidentiality and anonymity, all identifying information from interview transcripts were removed and participant demographic information was reported as a group. We refer to participants according to pseudonyms in the presentation of study results. This study was conducted in accordance with the ethical principles outlined in the Declaration of Helsinki (World Medical Association, 2013), and received ethical approval from the University of Ottawa Science and Health Sciences Research Ethics Board [H-02-20-5338].

## Results

### Summary of main themes

To comprehend MS caregiver resilience, we drew from participants’ illustrations and contemporary theories of resilience as a cyclical endurant process. Following analyses, a total of five themes emerged, including one major theme and four subthemes. Ultimately, the experiences shared by participants were captured in a major overarching theme, which extends beyond linear and segregated resilience trajectories: “resilience is a continuum of languishing to flourishing.” Collectively, the narratives of MS caregivers were subsumed within four smaller subthemes as a reflection of the complexity of resilience and its constituent conceptual pillars: “resilience resonates divergently,” “additive caregiving challenges,” “resilience resources originate from human connection,” and “adaptation begins with a learned mindset”.

Firstly, the subtheme of divergent resilience conceptions emerged from caregivers’ abstract understandings of resilience in their own words, as informed by their inimitable backgrounds. In reference to the component of adversity in resilience systems, the subtheme of dominant additive challenges refers to statements which reflected the overabundance of compounded and continuous challenges innate to the MS caregiving experience. As captured within the third subtheme, participants moved forward within a resilience continuum despite constant challenges by leveraging individual and community resources which arose from ubiquitous social connection as fundamentally interactive beings. To characterize the last subtheme, participants discussed ingenious adaptive processes that would not have been possible without their progressing wisdom and cultivated mentalities.

### Overarching theme: resilience is a continuum of languishing to flourishing

In the examined context, resilience cannot be portrayed conceptually as a fixed linear outcome or trajectory. This notion is supported by the transitory nature of the challenges inherent to MS caregiving, and reflects the unique characteristics of the disease (e.g., chronicity, progression, heterogeneity, unpredictability, polysymptomatic aetiology). Within the MS caregiver role, the presence of continuous struggles and accumulating losses places resilience along a fluid spectrum, determining caregivers’ future experiences of adversity and their health outcomes. Therefore, we conceptualize MS caregiver resilience within a cyclical paradigm.

In accordance with the three facets of resilience (i.e., an encounter with adversity, adaptation, and preserved well-being), this cycle operates synergistically with multiple influencing factors, including resilience resources and adaptation components (Windle & Bennett, 2011). In essence, the availability of individual, community, and societal resources informs the caregiver’s process of adaptation (Windle & Bennett, 2011). Collectively, these system factors contribute to caregivers’ degree of adjustment and healthy functioning, situated along a continuum of languishing to flourishing. Consequential adjustments exert a feedback function whereby they contribute to caregivers’ experiences of future encounters with hardship, encompassing subsequent appraisal and adaptive processes.

### Subtheme 1: resilience resonates divergently with MS caregivers

In their resilience definitions, most participants incorporated an encounter with adversity and referred to the concept of adaptation. A few participants described resilience as attaining equilibrium in mental state or the development of superior mental and physical well-being following confrontation with a problem. Participants’ definitions of resilience aligned with both *trait*-based and *process*-based resilience conceptualizations. With respect to trait-based definitions, Arthur described resilience as a partnered concept, stating that resilience is the “property of the person, and in this case the person you’re looking after.” Participants frequently described resilience with certain attributes such as perseverance, patience, and inner strength; some even associated resilience with the quality of being resolute, determined, and firmly rooted in intent. For example, Vivienne termed resilience as “a strength, maybe of character that you’re—you have some sense of determination.”

With that said, participants alluded to resilience as a more fluid construct, conceptualizing resilience as a flexible process, involving long-term grit and continuity. This notion tended to incorporate the role of emotions and their provisional nature within the resilience cycle:

Resilience […] is about bouncing back, I think, right? Allowing yourself to have those very real emotions, giving yourself that space to feel those very real reactions to things, and then saying, “OK, I can’t change that, let’s move on, let’s find a plan.” I think a lot of people think that resilience is about not being affected by things, but I don’t find that accurate. You know, I have to feel everything first and process it and then I can move forward with plans. (Tara)

I think people vacillate between having to get through a difficult physical problem and the emotional need to bear down and work through it. And prepare for it again in the next hour or the next day or next spring. (Daryl)

### Subtheme 2: dominant caregiving challenges are additive

#### Accumulating loss due to disease progression

Across all participants, emotions of grief were experienced because of the loss associated with the navigation of MS as an incurable disease possessed by a loved one. Emotions of grief originated from loss due to missed opportunities, advancing physical limitations, and the impact MS as a disease exerts on their relationship with the care-recipient. MS caregivers were grieving multiple connected losses from the past, present, and future. For instance, when asked about his emotional response to the deteriorating health of his wife with MS, Eli shared that “the biggest challenge of the whole experience is that you’re not grieving for a day or a week or a month, it’s for the whole thing. Because there’s always that change and […] you’re also grieving the loss to come.” Caregivers described MS as a series of losses and, in turn, an experience of continuous grievance for exacerbated losses:

[MS] is a loss of one thing after another after another after another. You know, the loss of being able to walk, you know the loss of being able to have a partner that can assist with household chores, that can assist with childcare, that can support you. (Laura)

For spousal caregivers, grief stemming from the unforeseen changes in their relationship and future together as a couple was discussed frequently. Unplanned futures were difficult for caregivers to accept:

I still every once in a while, have a bad day and I’ll just be down in the dumps because it’s not fair. Just things like that, we can’t go on a hike in the woods or we can’t buy that house within a matter of minutes, it’s taken us months and months to do everything. I still get upset by stuff like that. (Grace)

The primary challenge with the loss experienced by caregivers as a consequence of MS was that caregivers could never find solace in knowing whether their loss was complete or definite. Just as caregivers began to gradually adjust to losses spawned from their care-recipients’ latest disease progression or disability accumulation and accept the associated ramifications for their life and partnership, another form of complicated loss was just around the corner. The unpredictability and incompleteness of loss made it exceptionally difficult for MS caregivers to durably surmount caregiving challenges as, unlike in non-progressive disease contexts, another form of significant loss is likely imminent. This theme further supports reframing resilience within a cyclical paradigm, whereby resilient processes are characteristically indefinite within MS caregiver experiences.

#### Helplessness and obstacles of empowerment

Grief in caregivers coexisted with a sense of helplessness and a lamentation over a loss of control in the face on an unpredictable disease. These emotions were often intertwined with pity and frustration, as exhibited by William who shared that he was “frustrated that there isn’t a cure, frustrated that there’s no real treatment for the type of MS [his wife possesses],” and that “there isn’t anything better right now.” Feelings of powerlessness were typically heightened by difficulties linked to MS healthcare and research navigation, including a lack of definitive answers from healthcare providers, limited MS treatment options, and challenges related to accessing and coordinating support resources for the care-recipient. The limited accessibility of information and resources available to persons with MS and their families was a marked obstacle for caregiver empowerment. For instance, Tara recounted her experience with these shortcomings of the health system:

[The neurologist] didn’t give us any information on paramedical services that might be useful, like, physiotherapy, occupational therapy, things like that […] There are so many things that can add quality to life […] And it’s hard for me to find this information and these resources.

Due to its early age of onset and mild to advanced disability levels (Wallin et al., 2019), MS can impact families at all stages of life and in very different ways. The lack of appropriate support by disease stage increased caregivers’ sense of helplessness, contributing to the inability to obtain validating resources from community support services. Caregivers identified this unmet need and remarked that seeing more advanced stages of the disease can even be frightening:

The big issue for me with MS is there’s just absolutely no support. You know the only group in town for my husband is on a Friday morning and […] when he first got MS I brought him there and the people were so different in where they were at with the disease that he was just horrified with the progression in most of the people that were there. (Linda)

I haven’t found any local resources that I really think were appropriate for us, because we are relatively young and, like, it’s crazy, because there’s so many people out with MS or people that know people with MS, and there’s just not local groups that get together. (Tara)

Notably, barriers to accessing high-quality community support are far from ubiquitous. In truth, support access for persons with MS and their families is a function of numerous factors, including residing in urban versus rural settings, regional or local activism, access to transportation, other life commitments, and financial resources. This reality was frequently acknowledged by participants and coloured their recounts of interacting with community services.

#### Role intersection and threatened self-care

The most significant challenge experienced by caregivers was the threat their caregiving role posed for their ability to prioritize their own self-care requirements. Caregivers’ personal needs were overshadowed by the complex social, emotional, and physical needs of care-recipients. Sophia reflected on these complexities:

I completely lost track of my own personal life and bubble in that time [of diagnosis], because the focus was totally on [my husband]. I felt like I needed to try to keep everything in check, so that I could be there for him and not really acknowledge the impact that things had on me.

Other caregivers like Laura, acknowledged the impact negotiating a support role can have on caregiver wellness, and that some caregivers are subjected to problematic vulnerabilities:

We [caregivers] are so immersed in providing care for someone else so we have to recognize that we’re equally as needing and as valuable as worthy of care and we can’t get it from that partner, right? We can’t get that care from them. They’re not able to give it to us so we have to find it somewhere.

Furthermore, roles and responsibilities intersected to compromise caregivers’ capacity to practise self-care. Participants encountered hardships when trying to balance their caregiving role with other life roles such as their duties as a professional, community member, and parent, sibling, or child. For instance, Tara conceded that she felt overwhelmed by her intersecting caregiving roles in both home and professional settings:

All of this caregiving is kind of, like, piling up on each other, right? Having a baby, and then a partner that will need caregiving, and then I was working in a long-term care home all kind of at the same time.

### Subtheme 3: resilience resources originate from human connection

As participants sought to thrive within their roles, resilience resources at the individual and interpersonal level were most frequently discussed. Participants cited the importance of individual factors of mindfulness within their daily lives and empathy when approaching their support role. These factors typically possessed a protective function for caregiver-care-recipient relationship well-being, strengthening the quality of their relationships and preventing relational strain. Thus, even individual-based factors themselves permeated into relational and community ecological levels.

#### Mutuality, general social support, and community engagement

As a characteristic of the interpersonal relationship between the caregiver and care-receiver dyad, mutuality is a construct that denotes relationship quality (Park & Schumacher, 2014). Mutuality was a key interpersonal level resource shared between the caregiver and care-recipient that promoted resilience. Compassionate shared communication and honesty were crucial for relational well-being:

Make sure that there’s the open communication with the person that you’re caring for, that you can understand what the symptoms are and how they’re impacting them, so that you can work together to come up with a plan to help. (Serena)

As best I can, I try to see things from [my wife’s] perspective and try to approach it with empathy. I don’t always succeed at that, but at least I try to imagine what it’s like for her and see if there are ways that I could intercede or offer support or something that will help out. (Leroy)

Likewise, general social support was a core channel of community resilience. Participants shared beliefs that many of their hardships could not have been surmounted without the support of friends, family, neighbours, and co-workers. Caregivers benefited from various forms of support, but emotional support was particularly valuable. It was important that they had someone to confide in, regardless of whether that person was able to fully appreciate their struggles:

Mentally I need social connections, I need people to talk to […] it’s really difficult for people to understand, but they listen, and they do the best they can and that’s good enough right now. (Linda)

Having somebody else to bounce off, a balance board, just to talk through ideas or talk through different – I had a really good friend that her daughter was blind because of cancer. And she was a really good friend when [my husband] was first diagnosed too. (Grace)

While social connection is universally valuable, participants expressed that the relationships they shared with people who genuinely had some grasp of what they were going through were of the greatest importance. It was through these intentional, high-quality, and understanding relationships that caregivers were able to overcome some of the most weathered caregiving challenges. Despite the utility of community support, participants expressed at times that they found it difficult to ask for help. Still, Laura and other caregivers, unanimously agreed that reaching out to others in times of need was essential to not only care for the care-recipient, but also to care for themselves:

I resisted [asking for help] for a really long time and I think that’s part of why I got into a crisis […] I’ve had a good support system, but I think probably it was to my detriment that I didn’t reach out to some community groups earlier because that really did help […] That’s been another resource for me.

Community initiative and involvement, such as volunteering and religious group participation, was described by caregivers as a means of personal enrichment, sustaining well-being and providing a meaningful opportunity for temporary reprieve:

Staying involved helps me from getting depressed and controlling depression […] The social interaction, the groups that – you know, I organized the chess club and ran some chess tournaments and so on. That was an activity in the community and so on and that gave me a bit of a profile in the community and, you know, some social status which I think that sort of thing was very helpful. (Richard)

#### Engagement with professional societal networks

At the broader society level, caregivers seldom shared that they used societal resources beyond healthcare systems. Lorraine highlighted the role of the local health integration network (LHIN) services, sharing that the network had connected her with helpful healthcare professionals:

Look into as many services as you can find. Like, getting LHINs, like, help outside, physiotherapy, I think personal support workers coming in the house are number one.

Through connections facilitated by these services, caregivers were able to obtain additional help as they managed care-recipients’ changing disability levels. Trained external providers represented a neutral party that relieved potential sources of strain in the caregiver-care-recipient relationship as the MS care-recipients’ independence declined.

### Subtheme 4: adaptation begins with a learned mindset

Through processes of emotional management and compartmentalization, participants were able to draw on intrapersonal resources to develop a mindset conducive to adaptive processes. These adaptive processes facilitated favourable adjustment outcomes within the resilience cycle and influenced how caregivers responded to future encounters with adversity within their role. By compartmentalizing their reactions to unfavourable illness-related events and openly accepting the resultant limitations placed on the care-recipient and their life together as partners or family members, caregivers managed to continue to adaptively move forward:

There’s so much you learn you can’t control. So, don’t worry about it. There’s nothing I can do until it happens, so, we’ll just focus on what we can deal with. (Tracey)

Accept the limitations of the person that you’re caring for […] Accept that it will happen but don’t take it out on the partner […] You know the famous quote of ‘accept the things you can’t change and change the things you can’t’. And so if there is an action you can do then that’s fine, if there’s nothing you can do then you just have to accept the fact that that’s the way it is and move on and find other things to do. (William)

Caregivers’ accepting mentalities and sequential adaptation processes were influenced by individual and community resources. As their caregiving experience progressed and was matched by a deepening mindset of acceptance, participants commonly adopted a proactive approach, observing key indicators in MS care-recipients and sensitively recognizing their restrictions:

Learning what [my husband] was capable of was - and what is his limits, and when during the day. I find those are pretty consistent with his MS. He’s more tired in the mornings so he won’t do anything before 10 and then at seven he’s done. So I’m just figuring out how he is. We’ve learned to manage a lot of stuff. (Jolene)

Well so some ways I’ll adapt for my wife, you know obviously when she’s experiencing episode of flare ups, they will usually limit her physical stamina to do things or her, sometimes her physical strength. So, say chores around the house that are let’s say more laborious like vacuuming for example, […] we’ve adapted by sort of trading off the things that are harder for her to do. (Leroy)

Situational anticipation—that is the ability to read the care-recipient and prevent problematic situations before they arise—was a skill that caregivers developed over time. This skill contributed favourably to magnifying the resilience cycle and promoted positive adjustment consequences. This acquired approach was followed by high-efficacy problem-solving, including actions that preserved care-recipient autonomy, incorporated creative solutions, and tackled MS via lifestyle factors:

I’m always encouraging [my husband], let’s try to get a dish here you know like when he couldn’t reach the sink to spit anymore. OK, so you know you can still brush your teeth, we’re not going to give up on this, but here just hold the dish. (Laura)

About ten days before our wedding, [my husband] had a flare, and so it was just, okay, so these are the facts, this is what’s happening, and we just tried to come up with contingency plans of, like, if his legs get too weak, we can rent a wheelchair if we have to, or we can get a walker for him, and we had icepacks. (Sophia)

We looked back towards my partner’s lifestyle and experiences, diet, emotional, physical, mental status. Anything that could’ve been a contributing factor […] and we wanted to find some sort of pattern in terms of is it stress, is it a diet, is it environmental factors that kind of brings out these kinds of relapses so that we know that we can work with our everyday situation to prevent any of this stuff from happening […] We changed diets ASAP, we really looked at the broader picture of just our lifestyle. (Norah)

It was through these complex processes that participants were able to exhibit personal growth and preserve well-being, preparing them to adeptly rise above future caregiving challenges. In the absence of key resources and adaptive steps, participants were placed at risk of burnout and other deleterious outcomes, and the resilience cycle was temporarily disrupted.

## Discussion

To our knowledge, this is the first study to explore how MS family caregivers individually conceptualize and experience resilience. Our analysis constructed a cyclical model of resilience whereby ever-present challenges are overcome within a complex and continuous paradigm. We did not intend to determine *if* MS caregivers achieve resilience, but rather *how* they participate in the ubiquitous process. This focus aligns with contemporary assertions that resilience is made of ordinary rather than extraordinary processes; and is a natural process of caregiver adaptation (McKenna et al., 2022). Our findings suggest that MS caregivers possess comparable resilience levels to other caregiving populations (e.g., Lavretsky et al., 2010; Serra et al., 2018; Wilks & Vonk, 2008), but exhibit differing lived experiences of resilience.

Accordingly, caregivers expressed difficulties defining resilience and no consistent definition was observed. To echo current debate in the resilience literature, some caregivers perceived resilience as an individual trait, while others described it as a process. This discordance confirms that resilience is enigmatic both as an academic construct and when considered by community-dwelling caregivers. Interestingly, multiple caregivers associated resilience with emotional regulation. This finding is supported in literature involving general populations where emotional regulation and emotion-oriented coping strategies have been linked to building resilience (M. M. Smith et al., 2016; Tugade & Fredrickson, 2007). Beyond its presence in participants’ conceptualizations, references to emotional regulation also emerged within the resilience cycle. In the dataset, emotional regulation facilitated perspectives that incorporated acceptance and minimized disadvantageous reactive behaviours and could represent a gainful area to target as a means of harnessing resilience processes in MS caregivers.

As the catalyst component of the resilience cycle, participants identified diverse challenges within their caregiver role such as accumulating loss, a marked sense of helplessness exacerbated by deficiencies in community and health systems, and threatened self-care compounded by intersecting life roles. Within the ecological resilience framework, caregivers surmounted challenges through individual resources, such as cognitive empathy and daily mindfulness. Primarily, community resources originating from social connection were integrated into resilience pathways, consisting of caregiver-care-recipient mutuality, broad social support systems, and meaningful community participation.

The process of adaptation as a feature of resilience in MS caregivers proved to be highly nuanced; however, several patterns emerged. When discussing resilient experiences, participants shared that their adaptive decision-making processes were informed by an overarching mindset. Caregivers’ socially constructed mentalities were permeated by radical acceptance, emotional compartmentalization, and redefined appreciation. Overall, these mindsets prevented shared suffering from becoming debilitating for the caregiver as the disability of the care-recipient advanced, and enabled caregivers to steadily adapt in their helping role. Similarly, through open acceptance of care-recipients’ limitations, caregivers were motivated by unconditional love to persevere and compassionately alleviate suffering in their loved one with MS.

The clarity afforded by adaptive perspectives guided successive adaptation through empowerment; participants adopted a proactive approach and leveraged factors they could still control. Indeed, a proactive approach, preceded by situational anticipation, was a common theme among testimonies of adaptation. Caregivers learned to detect key indicators in care-recipients to better interpret their mental and physical state. This approach enabled carers to intervene before problematic consequences arose for the person with MS and prevented the need to turn to less effective reactive strategies. When caregivers felt empowered, their confidence and caring capabilities increased, which improved their quality of care and stimulated patterns for future action. Intriguingly, this process has been comparably identified in existing empowerment literature in family caregivers of older adults (Sakanashi & Fujita, 2017). Efficacious problem-solving action typically followed this anticipatory approach, whereby caregivers were able to minimize avoidable care-recipient suffering and preserve autonomy. In fact, interpersonal communication and caregiving practices that are autonomy supportive for the care-recipient have been identified to improve chronic illness outcomes (Stawnychy et al., 2021) and may confer benefits for both the caregiver and care-recipient. Thus, these actions permitted heightened adjustment and well-being benefits, which exerted a feedback function to prepare caregivers for future hardships along the resilience spectrum.

### Key interpretations and implications for practice

A central theme among challenges that mobilized resilience processes included the narrative of continuous loss and helplessness innate to witnessing a loved one endure a progressive disease. Research in other CNCs (e.g., acquired brain injury, dementia) has identified loss in caregivers as ambiguous (Nathanson & Rogers, 2021; ThØgersen & Glintborg, 2020). In the context of chronic illness, ambiguous loss refers to a situation where a loved one is physically present but psychologically absent, and this phenomenon, where loss is unclear or incomplete, affects both the care-recipient and caregiver (Boss & Yeats, 2014). Psychological absence in individuals with MS, as a central component of ambiguous loss, may occur due to cognitive and behavioural deficits in long-term memory, attention, information processing, and executive functioning (Preston et al., 2014). Furthermore, MS is an incurable illness with an unpredictable trajectory and is rife with ambiguity (Boss & Yeats, 2014); caregivers revealed that this genre of loss is often immobilizing and rendered them more prone to distress and relational conflicts (Boss & Yeats, 2014). With that said, empathy was a pivotal resilience resource used by caregivers to manage the loss associated with the mental instability exhibited by MS care-recipients. This tool enabled caregivers to not take care-recipients’ fluctuating psycho-behavioural states personally and prevented relational strain. These findings highlight the need for clinicians and other interventionists to seek to empower MS family caregivers as they experience ambiguous loss and chronic exposure to adversity, helping to preserve relational cohesion and strengthen resilience pathways via utilization of empathy.

To come to terms with ambiguous loss and alleviate feelings of helplessness, family and community processes have been shown to support resilient recovery (Masten, 2016). Meeting with other families experiencing similar loss aids in the process of labelling feelings and encourages acceptance (Boss & Yeats, 2014). However, in their quest for support, caregivers reported that appropriate MS support tailored to disability level of care-recipients were unavailable due to the mosaic of MS stages and clinical presentations. This shortcoming made it onerous for caregivers to access a critical resilience asset: to find comfort in others and connect with those who comprehended what they were truly going through in that moment. This impedance represents a crucial area in need of future resource and program development at community and societal levels to ensure that MS caregivers have access to suitable interpersonal supports which encourage resilient systems.

Threatened health-related self-care, whether due to role overload, lack of time for personal needs, or poor service coordination, was a key finding. The most robust obstacles adversely affecting health-promoting self-care were additive, such that their support role and unmet needs made it exceptionally challenging for caregivers to cope with life challenges such as parenthood, the death of a loved one, moving or relocating, and personal health issues. This finding is concerning because low engagement in self-care practices in family caregivers of persons with other chronic illnesses has been linked to increased anxiety, depression, poor quality of life, and other morbidities and appears to be the prerequisite for burden and crisis (Dionne-Odom et al., 2017; Oliveira et al., 2019).

Services to combat overshadowed caregiver needs were not always readily available, a reality reverberated by the minimal availability of resilience resources at the societal level. This finding indicates that systems at the structural and policy levels are offering insufficient access to important high-level resources including complementary health services and affordable respite care. Indeed, the inadequacy of support services accessibility and coordination for other family caregivers has been previously documented in the literature (Oliveira et al., 2019), particularly with respect to respite care and information awareness. Societal policies and programs should be targeted and developed to further enhance MS caregiver resilience and reduce risk. This assertion is supported by previous research in other caregiving populations which demonstrated that respite care is a significant resilience facilitator (Donnellan et al., 2015; Joling et al., 2016).

Several participants referred to individual and community factors that have enabled them to intentionally prioritize their personal needs despite the ever presence of obstacles of self-care. Despite the omnipresence of these challenges, caregivers manage them adaptively via the integration of available resources and continuous adjustment. Ultimately, this finding points to the importance of social context, namely community and social resources, for resilience cultivation—consistent with previous MS literature (Silverman et al., 2017). Still, the majority of existing caregiver interventions focused on individual clinical and sociodemographic variables rather than surrounding context. Correspondingly, our findings suggest that broad service and systems level factors, rather than individual and interpersonal factors, are the most deficient areas of the resilience cycle and warrant targeted research, clinical, and policy-level focus.

With improved consideration of contextual factors and how they impact caregiver resource availability and efficacy, asset development for more vulnerable MS caregiver populations that are difficult to reach (e.g., those in rural areas or those who experience socioeconomic disadvantages) may be realized. Such populations ought to be targeted via innovative intervention mechanisms which favour telemedicine approaches to minimize accessibility barriers and promote continuity of caregiver support services (Dal Bello-Haas et al., 2014).

### Strengths and limitations

This study contributes much that is novel to the MS caregiving literature. By focusing on the distinct and understudied MS caregiver population, we offer an unprecedented perspective through which we examined resilience. Via in-depth individual interviews of a robust qualitative sample size (Boddy, 2016), we identified *how* MS caregivers experience resilience and pinpointed key challenges, resilience resources, and adaptive mechanisms. Additionally, we sampled MS caregivers of care-recipients with a range of disability levels to ensure sufficient representation of the caregiving experience within a multi-course disease with moderate to severe disability presentations.

Although this study obtained valuable insight into MS caregivers’ experiences of resilience, it was not accomplished without limitations. Firstly, as qualitative researchers mindful of potential sources of bias, it is possible that more resilient caregivers were more inclined to participate in this genre of research. Thus, experiences of resilience oppositions may not have been as readily discussed. Furthermore, because participants were managing diverse life stages and possessed various needs, it was less possible to establish key areas of resource deficiencies and successes. As such, longitudinal experience-based research may offer more comprehension of the relationship between transient forms of adversity at progressing life stages and cyclical resilience.

Additionally, this research was conducted in a sample of Canadian MS family caregivers with minimal diversity. As a product of little geographic distribution and limited ethnic and cultural diversity within this sample, the influence of regional and sociodemographic factors on perceptions and experiences of resilience could not be determined. Still, this dataset was indicative of the importance of some factors for resilience development, such as age, biological sex, care-recipient disease severity, socioeconomic status, and employment status, and this may represent an interesting avenue for future research. Moreover, most of our sample consisted of spousal caregivers and other family caregiving relationships were underrepresented. Thus, the patterns observed in these data may be more reflective of the spousal MS caregiver experience. Lastly, interviews were centred on dyadic relationships between caregiver and care-recipient, viewed through caregivers’ individual perspectives. Although mutuality and broader community social networks are discussed in the themes presented herein, we did not directly explore the implications of family functioning for caregiver resilience development. However, it is worth noting that the “family” of individuals living with disabilities often extends far beyond biological relationships (McDaniel & Pisani, 2012), and may have been indirectly captured within our community level themes composed of heterogenous relationships at different life stages. With that said, family dynamics are becoming increasingly integrated into the broader caregiving and disability fields (McDaniel & Pisani, 2012), and future exploration of MS caregiver resilience specifically in the context of family systems is warranted.

## Conclusion

In MS caregivers, we demonstrate by applying the ecological framework (Windle & Bennett, 2011) that the process of resilience is cyclical and encompasses complex antecedents, facilitators, and mechanisms of adaptation. Participants did not concur on a single resilience conceptualization, although their testimonies support that resilience is a normative process. Our analyses yielded a cyclical model of resilience headed by significant challenges of ambiguous and perpetual loss, powerlessness, and threatened self-care. As modulators of resilience, resources emerged mostly at the individual and community levels. Adaptive pathway factors commenced with a mindful and accepting mindset, followed by empowered approaches and problem-solving action. Ultimately, through these intricate pathways, adjustment and well-being were achieved which cyclically informed future responses to challenges. The experiences of resilience within this population emerged in spite of inadequate resources at the societal level, and points to the need for better support stratifications and development of interventions that address contextual factors for MS caregivers.

## Supplementary Material

Supplemental Material

## Data sharing

Data is available upon reasonable request.
