# Supplementary material for: A continuum of languishing to flourishing: exploring experiences of psychological resilience in multiple sclerosis family caregivers
Source: Int J Qual Stud Health Well-being. 2022 Nov 4;17(1):2135480. doi: 10.1080/17482631.2022.2135480 (PMC9645274; doi:10.1080/17482631.2022.2135480)
Supplement: Supplemental Material [file ZQHW_A_2135480_SM9591.docx]

**Appendix 1**

**Interview Guide**

**Consent process**

*Begin recording once you have gone through the consent process. Begin recording for verbal consent. Pause recording once verbal consent has been stated.*

**Introduction**

I would like to thank you for participating in this interview to explore how MS care partners, like you, manage the challenges of caregiving, especially during difficult times like the COVID-19 pandemic. As I ask you to describe your opinions and experiences, please keep in mind that there are no right or wrong answers to these questions, since people have a lot of different views on these topics. I’m simply interested in what you think about these different issues. I don’t know exactly what it’s like to “be in your shoes” or to deal with the challenges that care partners like yourself are confronted with every day, so I am looking forward to learning more about these experiences from your perspective. Thank you for giving me your time. This interview should take around 45 minutes to one hour.

Before we begin, do you have any questions pertaining to the study?

*Answer any questions*

If at any time you have questions or something that I say is not clear, please let me know and I’ll try to clarify.

*Restate permission to record the interview.*

Warm-up/building rapport

Before we start talking in detail about the topic, I would like to first know a little about you. Pretend that you are someone close to you, like your best friend or family relative, and you are talking to someone who has never met you. Describe to that person who you are. Give as much or as little detail as you feel comfortable telling me—this is just a way for me to get to know you better so that I will know how to best ask you the other questions in this interview.

Thanks for sharing a little bit about what’s going in your life right now. Now, I’d like for us to begin talking about resilience, caregiving, and the impact of the current pandemic on your life as a care partner.

*Begin recording at this point (let participant know that recording is starting)*

Section 1: Resilience

1. What does having resilience mean to you?
   - Probes:
     - What comes to your mind when you hear this word?
     - If you were to explain resilience to someone else, how would you explain it?
2. Tell me about some of the challenging situations that you have experienced as a care partner?
   - Probe:
     - Tell me about some of the challenging situations that you have experienced as a care partner in relation to the COVID-19 pandemic?
     - Has anyone in your immediate household been diagnosed with COVID-19?
3. How did you initially react when these things (mentioned above) happened?
   - Probe:
     - How did you react to situations related specifically to COVID-19?
4. How have you adapted your caregiving protocols, plans, and activities over the years?
   - Probes:
     - How have your caregiving skills/strategies changed in response to the current pandemic?
5. If you were talking with other care partners of people with MS, what would you tell them with respect to what has helped you to develop resilience during this pandemic?
   - Probes:
     - How has your community (e.g., local programs or services) helped you to develop resilience?
     - How has your social network (e.g., friends or family) helped you to develop resilience?
     - How about personal factors or personality characteristics about yourself that have helped you to develop resilience?
6. What factors have hindered you from developing resilience during this pandemic?

Section 2: Health and Wellbeing

*Now we will be moving on to questions related to your personal wellbeing throughout the course of this pandemic.*

1. How did the COVID-19 pandemic impact your ability to engage in health behaviours (e.g., healthy eating, limiting substance use, maintaining an active lifestyle, positive social engagement)?
2. What is the most challenging element of this pandemic for maintaining your health and wellbeing (physical, mental, or emotional)?
   - Probe:
     - How did these challenges impact your ability to be a care partner?
     - What new or alternative strategies (e.g., remote online tools, support lines, recommendations) have you used during this time in order to maintain your health and wellbeing?

Section 3: Disaster Preparedness

*Now we are going to explore another dimension related to your resilience experience during this pandemic.*

1. How prepared did you feel for a pandemic of this nature and its impact on your ability as a care partner?

Closing questions

Is there anything else that you would like to tell me about your experience of stress or difficulties as a care partner during this pandemic?

Are there any experiences of resilience as a care partner that you want to elaborate on?

Thank you for sharing your insights with me today. As you know, the purpose of this interview is to generate further insights about resilience among care partners of people with MS, and how care partners cope with challenging and stressful situations, like the COVID-19 pandemic. The insights that you have shared with me with today may inform the development of programs to enhance and sustain resilience among care partners of people with MS.
